# Empirical evidence for outcome reporting bias in randomized clinical trials of acupuncture: comparison of registered records and subsequent publications

**DOI:** 10.1186/s13063-014-0545-5

**Published:** 2015-01-27

**Authors:** Chun-Xiang Su, Mei Han, Jun Ren, Wen-Yuan Li, Shu-Jin Yue, Yu-Fang Hao, Jian-Ping Liu

**Affiliations:** Center for Evidence-Based Chinese Medicine, Beijing University of Chinese Medicine, 11 Bei San Huan Dong Lu, Chaoyang District, Beijing, 100029 China; School of Nursing, Beijing University of Chinese Medicine, Chaoyang District, Beijing, 100102 China

**Keywords:** Selective outcome reporting, Outcome reporting bias, Publication bias, Trial registration, Acupuncture, Randomized clinical trials

## Abstract

**Background:**

Outcome reporting bias has received widespread recognition and been considered to pose two threats to the validity of clinical decision making because they overestimate the effect of treatments or distort the results of trials. However, the problem of outcome-reporting bias has not been systematically studied among randomized clinical trials of acupuncture. Our objectives were to evaluate the consistency between the registered records and subsequent publications with respect to outcomes and other data as well as to determine whether outcome-reporting bias favors significant primary outcomes.

**Methods:**

A systematic search of 15 registries was conducted from their inception to January 2014 to identify randomized clinical trials on acupuncture for which the status was listed as ‘completed.’ The subsequent publications were retrieved by searching PubMed and three Chinese databases. Basic characteristics and the registration information were extracted from the registered records and publications. We performed comparisons regarding primary outcomes and other data between the registered records and subsequent publications to assess the consistency and selective outcome reporting.

**Results:**

Eighty-eight trials on acupuncture with 96 published reports were identified. Only 19.3% (17/88) were registered before the start of the trial, suggesting prospective registration. The trial registration number was unavailable in 36 published reports (37.5%). A comparison of registered and published primary outcomes could be conducted in 71 publications (74.0%), and the inconsistency of the primary outcomes was identified in 45.1% (32 of 71); 71.4% (15 of 21) had a discrepancy that favored statistically significant primary outcomes, while 28.6% (6 of 21) favored nonsignificant primary outcomes. Furthermore, the other inconsistencies between the registry records and subsequent publications involved the inclusion criteria (54.7%), exclusion criteria (47.9%) and controls (22.9%).

**Conclusions:**

We find that prospective registration for randomized clinical trials on acupuncture is insufficient, selective outcome reporting is prevalent, and the change of primary outcomes is intended to favor statistical significance. These discrepancies in outcome reporting may lead to biased and misleading results of randomized clinical trials on acupuncture. To ensure publication of reliable and unbiased results, further promotion and implementation of trial registration are still needed.

**Electronic supplementary material:**

The online version of this article (doi:10.1186/s13063-014-0545-5) contains supplementary material, which is available to authorized users.

## Background

Outcome reporting bias in a clinical study is defined as the selection of a subset of the original variables recorded on the basis of the significance and direction of the results [1]. It distorts the results of trials and leads to a reduction in the strength of evidence for conducting systematic reviews and developing clinical practice guidelines [2-5].

Prospectively trial registration in international clinical trial registries before participant enrollment is one of the main ways to potentially reduce selective outcome reporting and further outcome reporting bias [6,7]. The International Committee of Medical Journal Editors (ICMJE) announced a policy of requiring investigators to register clinical trials in a clinical trial registry before participant enrollment as a prerequisite for publication in 2004. This policy took effect in 2005 and is applicable to any clinical trial beginning enrollment after 1 July 2005 [8]. The World Health Organization formally established the International Clinical Trials Registry Platform (ICTRP; http://www.who.int/ictrp/en/) in 2004 to push for clinical trial registration. Furthermore, several peer-reviewed journals, such as *The Lancet* and *Trials*, publish study protocols to achieve transparency regarding performance and reporting of clinical trials.

Outcome reporting bias has received much attention. Many empirical studies evaluating the consistency concerning outcomes between the protocol or trial registry entry of randomized clinical trials (RCTs) and subsequent publications have demonstrated that outcome reporting bias is prevalent and favors statistically significant results [9-16]. A recent study focusing on RCTs of traditional Chinese medicine (TCM) showed discrepancies were identified in primary outcomes (29%) and safety reporting (28%) when comparing registered records with their subsequent publications [17]. However, the problem of outcome-reporting bias has not been systematically studied among RCTs of acupuncture.

Our objectives were to evaluate the consistency between the registered records and subsequent publications with respect to outcomes and other data as well as to determine whether outcome-reporting bias favors significant primary outcomes.

## Methods

### Inclusion criteria

We included RCTs on acupuncture alone or in combination with other interventions that were registered in trial registries. The current status was limited to ‘completed’ in the registered records. Acupuncture was defined as a collection of procedures involving penetration of the skin with needles manipulated by the hands or by electrical stimulation to stimulate certain points on the body [18]. We excluded phase 1 trials.

### Search and selection of trials

A systematic search of 15 major international trial registries was conducted using the search terms “acupuncture” or “electroacupuncture” or “auriculotherapy” from their inception to January 2014. Trial registries included the Australian New Zealand Clinical Trials Registry (ANZCTR), Brazilian Clinical Trials Registry (ReBec), Chinese Clinical Trial Register (ChiCTR), Clinical Research Information Service (CRiS) public of Korea (KCT), ClinicalTrials.gov, Clinical Trials Registry-India (CTRI), Cuban Public Registry of Clinical Trials (RPCEC), EU Clinical Trials Register (EU-CTR), German Clinical Trials Register (DRKS), International Standard Randomized Controlled Trial Number Register (ISRCTN), Iranian Registry of Clinical Trials (IRCT), Japan Primary Registries Network (JPRN), Pan African Clinical Trial Registry (PACTR), the Netherlands National Trial Register (NTR), and Sri Lanka Clinical Trials Registry (SLCTR). If trials were listed as ‘completed’ in the registered records, the full texts of subsequently published articles were retrieved by searching PubMed without a language limit and three Chinese electronic bibliographic databases including China National Knowledge Infrastructure (http://www.cnki.net), Chinese Biomedical Database (http://sinomed.imicams.ac.cn/index.jsp), and Chinese VIP Information (http://vip.hbdlib.cn/index.asp) using the trial registration number, registered trial title, and investigator name. Identified articles were checked against the target registered records to determine whether they were a correct match. For each trial, all published articles reporting the final results were included. Abstracts and reports of preliminary results were excluded.

### Data extraction

Two authors (Su CX, Han M, Ren J, or Li WY) independently extracted data using a standardized, piloted data extraction form. The form was modified based on the previous research [17]. We collected the following information reported in trial registries and published articles: trial design, sample size, participants and diseases/conditions, interventions, controls, primary and secondary outcomes along with the time frame of assessment, inclusion and exclusion criteria, and funding. For each registered trial, we also extracted the date registered, start and end dates of participant enrollment/the trial, sponsors, and country of origin. Publication year and language, journal name, registered number, and *P* values for all the outcomes were also extracted from each subsequently published report. The 2013 impact factor of journals where registered trials were published was sought. ‘Instructions for authors’ of the included journals was checked for whether the registration of the trial and reporting of the trial’s registration number were required in the published report. The “history of changes” in the registries was tracked to determine whether the registered information had been changed since the initial registration. Conditions were classified according to International Statistical Classification of Diseases and Related Health Problems, 10^th^ Revision (ICD-10) (http://apps.who.int/classifications/icd10/browse/2010/en; last accessed on 27 January 2014).

### Data analyses

Methodological quality of registered records and subsequent publications was assessed independently by two authors (Su CX, Ren J, or Li WY) using the Cochrane Collaboration risk of bias tool, which included generation of the allocation sequence, allocation concealment, blinding, incomplete outcome reporting, selective outcome reporting, and other risk of bias [19]. Other risk of bias was assessed based on the estimated sample size, explicit inclusion and exclusion criteria, and the risk of funding bias.

Two authors (Su CX, Ren J, or Li WY) evaluated the consistency and selective outcome reporting regarding the trial design, methodology, and outcomes from the registered records and subsequently published articles. The disagreements were resolved by consensus with a third party (Liu JP). We defined inconsistency as “when the items in the trial registry were not the same as those in the subsequent publications” [17]. If over 20% discrepancy was identified between the sample size in the registered trials and published reports, we considered it inconsistent [19]. Selective outcome reporting was defined as “when the primary outcome specified in the published articles was different, or changed from those defined in the trial registry, or the timing of assessment of the registered and published primary outcomes differed” [20,21].

A discrepancy was considered to favor statistically significant results (*P* < 0.05) if a new statistically significant primary outcome was described in the published articles or if a nonsignificant primary outcome was defined as a nonprimary outcome in the published articles [10,21].

Data were presented as counts, percentage and frequency for categorical variables. Proportions were compared by the *χ*2 test. *P* < 0.05 was considered statistically significant. SAS version 9.1 (SAS Institute Inc, Cary, NC) was applied for statistical analysis.

## Results

**Figure 1:**
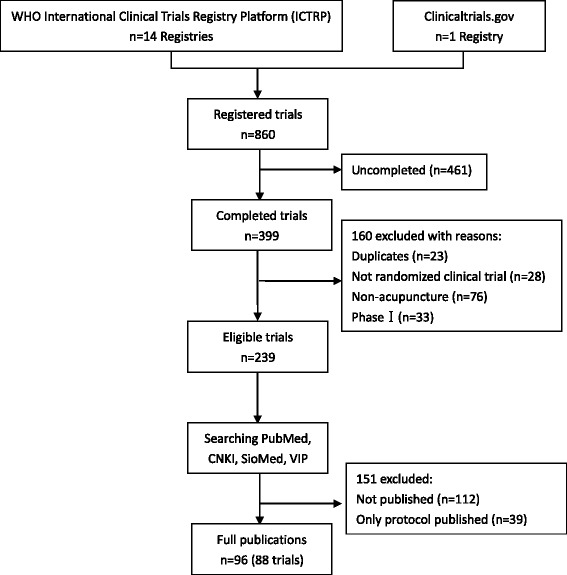
**Flow diagram of study selection.**

Of 860 registered acupuncture trials retrieved, 399 indicated their current status as ‘completed’. After excluding 160 registered trials because of duplicates or being non-RCTs, non-acupuncture and phase I, we searched subsequent publications for 88 of the 239 registered trials (36.8%). The 88 trials produced 96 publications reporting study outcomes, among which all publications were in English (Figure 1).

Of the 88 included trial protocols, 65 (73.9%; 72 articles) were from western countries; 23 (26.1%; 24 articles) were from eastern countries, of which only 7 were performed in mainland China (excluding Hong Kong and Taiwan). The included trial protocols were registered in eight registries and the majority was registered in either clinicaltrials.gov (43/88, 48.9%) or ISRCTN (36/88, 40.9%). The first trial was registered in 2001. Only 19.3% (17/88) were registered before the start of the trial, suggesting prospective registration. The remaining 71 trials were registered after the study began, even after the completion of the study. The proportion of retrospective registration was similar for trials from western countries (54/65, 83.1%) and eastern countries (17/23, 73.9%; *P* = 0.33). When comparing trials starting before 1 July 2005 (36/40, 90.0%), the proportion of retrospective registration was higher than that in trials starting after 1 July 2005 (35/48, 72.9%; *P* = 0.04).

**Figure 2:**
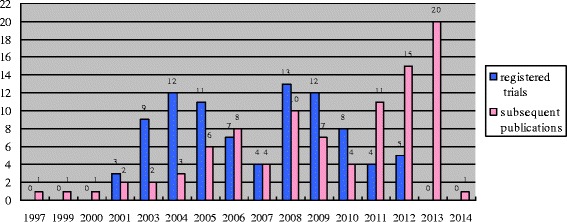
**Number of included randomized clinical trials on acupuncture in the trial registry and subsequent publication.**

Ninety-six articles were published in 49 journals from 1997 to 2014 (Figure 2). Seven (7.3%) were published in alternative and complementary journals. Sixteen (16.7%) were published in journals whose 2013 impact factor was high than 10 (Additional file 1: Table S1). Twenty-seven journals (48 articles; 50.0%) provided guidance on trial registration and reporting of the registration number in their instructions for the author. Of 36 articles (37.5%) without description of the registration number, 50.0% (18/36) were published in journals requiring the reporting of the registration number.

The characteristics of included articles are presented in Table 1. The majority of the included articles were parallel group (92, 95.8%), single center (55, 57.3%), two armed (59, 61.5%), and sponsored by a university, government or hospital (77, 80.2%). The five most frequent conditions based on ICD-10 were musculoskeletal (26.1%), nervous (19.3%), mental and behavioral disorders (17.0%), respiratory conditions (5.7%) and neoplasms (5.7%; Table 2). Manual acupuncture and electro-acupuncture were reported in 80 (83.3%) and 16 (16.7%) articles, respectively. Interventions in the treatment groups involved acupuncture alone in 70 (72.9%) articles or acupuncture combined with other interventions in 26 (27.1%) articles. There was considerable variation in the control group. Fifty-one articles (53.1%) applied sham/placebo acupuncture; other commonly used controls involved western medicine (26.0%), no intervention (9.4%) and non-pharmaceutical interventions (9.4%). A clear description of the primary outcome and secondary outcome was provided in 71 (74.0%) and 60 articles (62.5%), respectively. However, safety and health-economic outcomes were only reported in 38 (39.6%) and 5 articles (5.2%), respectively.

**Table 1:** **Characteristics of the included articles**

**Item**	**Total**	**Western countries**	**Eastern countries**
	***n*** **(%) = 96**	***n*** **(%) = 72**	***n*** **(%) = 24**
**Study registration**			
After the trial began	76 (79.2)	58 (80.6)	18 (75.0)
Before the trial began	20 (20.8)	14 (19.4)	6 (25.0)
**Study design**			
Parallel	92 (95.8)	68 (94.4)	24 (100.0)
Crossover	4 (4.2)	4 (5.6)	0 (0.0)
**Study center**			
Single	55 (57.3)	40 (55.6)	15 (62.5)
Multiple	41 (42.7)	32 (44.4)	9 (37.5)
**Number of arms**			
Two	59 (61.5)	46 (63.9)	13 (54.2)
Three	29 (30.2)	22 (30.6)	7 (29.2)
Four	8 (8.3)	4 (5.5)	4 (16.7)
**Funding source**			
No funding	14 (14.6)	13 (18.1)	1 (4.2)
University	37 (38.5)	25 (34.7)	12 (50.0)
Hospital	13 (13.5)	7 (9.7)	6 (25.0)
Government	27 (28.1)	23 (31.9)	4 (16.7)
Private non-profit	1 (1.0)	1 (1.4)	0 (0.0)
Other source	4 (4.2)	3 (4.2)	1 (4.2)
**Type of acupuncture**			
Manual acupuncture	80 (83.3)	64 (88.9)	16 (66.7)
Electro-acupuncture	16 (16.7)	8 (11.1)	8 (33.3)
**Intervention**			
Acupuncture used alone	70 (72.9)	51 (70.8)	19 (79.2)
Acupuncture combined with other interventions*	26 (27.1)	21 (29.2)	5 (20.8)
**Control†**			
Placebo/sham-acupuncture#	51 (53.1)	37 (51.4)	14 (58.3)
Western medicine	25 (26.0)	18 (25.0)	7 (29.2)
No intervention	9 (9.4)	7 (9.7)	2 (8.3)
Non-pharmaceutical interventions	9 (9.4)	9 (12.5)	0 (0.0)
Conventional therapy	7 (7.3)	7 (9.7)	0 (0.0)
Acupuncture	6 (6.3)	4 (5.6)	2 (8.3)
Waiting list	4 (4.2)	3 (4.2)	1 (4.2)
Chinese herbal medicine	1 (1.0)	0 (0.0)	1 (4.2)
**Specified primary outcome**	71 (74.0)	50 (69.4)	21 (87.5)
**Specified secondary outcome**	60 (62.5)	42 (58.3)	18 (75.0)
**Safety come**	38 (39.6)	24 (33.3)	12 (50.0)
**Health-economic outcome**	5 (5.2)	5 (6.9)	0 (0.0)
**Random sequence generation**			
Random number table	4 (4.2)	4 (5.6)	0 (0.0)
Computer random number generator	69 (71.9)	46 (63.9)	23 (95.8)
Minimization	2 (2.1)	2 (2.8)	0 (0.0)
Not reported	21 (21.9)	20 (27.8)	1 (4.2)
**Allocation concealment**			
Opaque sealed envelope	23 (24.0)	19 (26.4)	4 (16.7)
Central allocation	28 (29.2)	24 (33.3)	4 (16.7)
Sealed/opaque envelope	7 (7.3)	5 (7.0)	2 (8.3)
Not reported	38 (39.6)	24 (33.3)	14 (58.3)
**Blinding**			
Single-blinded	37 (38.5)	26 (36.1)	11 (45.8)
Blinding to participants	21 (21.9)	16 (22.2)	5 (20.8)
Blinding to personnel	3 (3.1)	2 (2.8)	1 (4.2)
Blinding to outcome assessor	13 (13.5)	8 (11.1)	5 (20.8)
Double-blinded	28 (29.2)	19 (26.4)	9 (37.5)
Blinding to participants and personnel	10 (10.4)	9 (12.5)	1 (4.2)
Blinding to participants and outcome assessors	18 (18.8)	10 (13.9)	8 (33.3)
Blinding to personnel and outcome assessors	0 (0.0)	0 (0.0)	0 (0.0)
Triple-blinded	4 (4.2)	3 (4.2)	1 (4.2)
Open	15 (15.6)	11 (15.3)	4 (16.7)
Not reported	12 (12.5)	11 (15.3)	1 (4.2)
**Incomplete outcome reporting**	33 (34.4)	28 (38.9)	5 (20.8)
**Sample size estimation**	34 (35.4)	22 (30.6)	12 (50.0)
**Explicit inclusion criteria**	94 (97.9)	71 (98.6)	23 (95.8)
**Explicit exclusion criteria**	82 (85.4)	60 (83.3)	22 (91.7)

Note: *Other interventions included western medicine in 18 articles, conventional therapy in 2 articles, non-pharmaceutical interventions in 4 articles, Chinese herbal medicine in 1 article and placebo drug in 1 article.

†Thirty-seven (38.5%) articles reported three or four arms in one study.

#Placebo acupuncture was conducted by using Streitberger placebo needles, which have a blunt tip. The needle retracted inside its handle when its tip touched the skin rather than penetrating the skin. Sham-acupuncture refers to nonspecific points, mock acupuncture/electro-acupuncture, mock transcutaneous electrical nerve stimulation, shallow needling and minimal acupuncture

**Table 2:** **Conditions based on the ICD-10 classification treated by acupuncture**

**Disease/conditions (ICD-10 codes)**	**No. of trials (%)**	**Western countries**	**Eastern countries**
		***n*** **(%)**	***n*** **(%)**
M00-M99 Diseases of the musculoskeletal system and connective tissue	23 (26.1)	19	4
G00-G99 Diseases of the nervous system	17 (19.3)	12	5
F00-F99 Mental and behavioral disorders	15 (17.0)	10	5
J00-J99 Diseases of the respiratory system	5 (5.7)	3	2
C00-D48 Neoplasms	5 (5.7)	5	0
E00-E90 Endocrine, nutritional and metabolic diseases	4 (4.5)	3	1
N00-N99 Diseases of the genitourinary system	4 (4.5)	1	3
O00-O99 Pregnancy, childbirth and the puerperium	4 (4.5)	4	0
K00-K93 Diseases of the digestive system	3 (3.4)	3	0
R00-R99 Symptoms, signs and abnormal clinical and laboratory findings, not elsewhere classified	3 (3.4)	3	0
A00-B99 Certain infectious and parasitic diseases	1 (1.1)	1	0
H60-H95 Diseases of the ear and mastoid process	1 (1.1)	0	1
Health	1 (1.1)	0	1
L00-L99 Diseases of the skin and subcutaneous tissue	1 (1.1)	0	1
S00-T98 Injury, poisoning and certain other consequences of external causes	1 (1.1)	1	0
Total	88 (100)	65 (73.9)	23 (26.1)

Seventy-five subsequent publications (78.1%) provided detailed information on the generation of allocation sequences with the most use of a computer random number generator. Fifty-eight articles (60.4%) provided details on allocation concealment. Of 69 articles (71.9%) providing information on blinding, 37 applied single blinding, 28 double blinding, and 4 triple blinding. However, only 3.4% (3/88), 2.3% (2/88) and 61.4% (54/88) of the registered records provided information on the generation of an allocation sequence, allocation concealment and blinding, respectively. Incomplete outcome reporting occurred in 33 (34.4%) articles. The sample size of the 96 articles ranged from 10 (pilot trials) to 960, with the majority (88.6%) of sample sizes between 20 and 500 per trial (Additional file 2: Table S2). Only 34 articles (35.4%) carried out a sample size calculation. All registered records lacked information on sample estimation. The majority of the registered records and the subsequent publications clearly described the inclusion and exclusion criteria.

A comparison of registered and published primary outcomes was impossible in 25 articles (26.0%) because outcomes were not specified as primary outcome or secondary outcome in the subsequent publications. Of the remaining 71 articles, the primary outcomes of 32 articles (45.1%) were inconsistent with those specified in registered records (Table 3). The discrepancies involved a registered primary outcome omitted in the published article (22/71, 31.0%), an absent primary outcome in the registry defined as a primary outcome in the published article (9/71, 12.7%), a published primary outcome registered as a secondary outcome (1/71, 1.4%), a registered primary outcome defined as a secondary outcome in the published article (15/71, 21.1%) and different timing of assessment of the primary outcome in the published article and the registry (12/71, 16.9%). Fourteen articles had two reasons for the difference in primary outcomes, five articles had three reasons for the difference in primary outcomes, and one article had four reasons for the difference in primary outcomes. This inconsistency was also addressed in 13 articles published in journals requiring trial registration; the proportion of inconsistency was lower than those published in journals not requiring trial registration [13 of 38 (34.2%) vs. 19 of 33 (57.6%); *P* = 0.048]. Five of 13 articles were published in a high-impact-factor journal (IF > 10). When comparing trials from western countries with trials from eastern countries, the proportion of this discrepancy showed no significant difference [6 of 21 (28.6%) vs. 26 of 50 (52.0%); *P* = 0.07].

**Table 3:** **Proportion of discrepancies in the specified primary outcomes between registered trials and published articles on acupuncture and discrepancies favoring statistically significant results**

**Discrepancy in published articles relative to registered trials**	**Total**	**Western countries**	**Eastern countries**
	***n*** **= 71 (%)**	***n*** **= 50 (%)**	***n*** **= 21 (%)**
**Articles with different primary outcomes in the trial registration and the published article**	32 ^a^(45.1)	26 ^b^(52.0)	6 ^c^(28.6)
Registered primary outcome omitted in published articles	22 (31.0)	18 (36.0)	4 (19.0)
An absent primary outcome in the registry defined in the published article	9 (12.7)	7 (14.0)	2 (9.5)
A published primary outcome registered as a secondary outcome	1 (1.4)	1 (2.0)	0 (0.0)
A registered primary outcome defined as a secondary outcome in the published article	15 (21.1)	13 (26.0)	2 (9.5)
Different timing of assessment of the primary outcome	12 (16.9)	8 (16.0)	4 (19.0)
**Discrepancies in primary outcome favoring statistically significant results** ^**d**^	32	26	6
Yes	15 (46.9)	13 (50.0)	2 (33.3)
No	6 (18.8)	4 (15.4)	2 (33.3)
Impossible to conclude	11 (34.3)	9 (34.6)	2 (33.3)

^a^Fourteen articles had two reasons for the difference in primary outcome; five articles had three reasons for the difference in primary outcome; one article had four reasons for the difference in primary outcome.

^b^Eleven articles had two reasons for the difference in primary outcome; five articles had three reasons for the difference in primary outcome.

^c^Three article had two reasons for the difference in primary outcome; one article had four reasons for the difference in primary outcome. Compared with articles from western countries: *P* = 0.07.

^d^A discrepancy in primary outcome was said to favor statistically significant results when a new, statistically significant primary outcome was introduced in the article or when a statistically nonsignificant primary outcome was defined as a nonprimary outcome in the published article.

Furthermore, 81.7% (138/169) registered primary outcomes were clinically relevant outcomes (patient-centered outcomes). Among them, 22.5% (31/138) clinically relevant outcomes from the 15 registered trial records were missing in the final publications, and 9.4% (13/138) clinically relevant outcomes from 11 registered records had been changed from the original primary outcome to the secondary outcome in the publications.

Of the 32 articles with inconsistency in primary outcomes between the published article and registry, the influence of this inconsistency could only be evaluated in 65.6% (21 of 32). Among them, 15 of 21 (71.4%) had discrepancies that favored statistically significant primary outcomes, while 6 of 21 (28.6%) favored nonsignificant primary outcomes.

Comparing the 88 registered records with their 96 subsequent publications, the other inconsistencies were identified in the inclusion criteria (54.7%), exclusion criteria (47.9%) and controls (22.9%) (Table 4).

**Table 4:** **Comparison of methodological components between registered records and subsequent publications of randomized clinical trials on acupuncture**

**Items**	**Total inconsistency rate (%)**	**Inconsistency rate of trials from western countries (%)**	**Inconsistency rate of trials from eastern countries (%)**
Study design^1^	7.0	10.5	0.0
Arms^2^	11.5	12.5	8.3
Intervention^3^	8.4	11.3	0.0
Control^2^	22.9	26.4	12.5
Sample size^3^	22.1	28.2	4.2
Inclusion criteria^3^	54.7	57.7	45.8
Exclusion criteria^4^	47.9	54.2	34.8
Generation of allocation sequences^5^	0.0	0.0	0.0
Allocation concealment^6^	0.0	0.0	0.0
Blinding of participants^7^	16.7	20.0	10.0
Blinding of personnel^8^	18.0	19.5	15.0
Blinding of outcome assessors^9^	22.8	21.6	25.0

^1^A comparison was available in 57 articles, of which 38 were from western countries.

^2^A comparison was available in 96 articles, of which 72 were from western countries.

^3^A comparison was available in 95 articles, of which 71 were from western countries.

^4^A comparison was available in 71 articles, of which 48 were from western countries.

^5^A comparison was available in 3 articles, of which 1 were from western countries.

^6^A comparison was available in 2 articles, of which 1 were from western countries.

^7^A comparison was available in 60 articles, of which 40 were from western countries.

^8^A comparison was available in 61 articles, of which 41 were from western countries.

^9^A comparison was available in 57 articles, of which 37 were from western countries.

The ‘history of changes’ was available in 44 registered trial records (44/88, 50.0%) from six registries. After the initial registration, a change of primary outcomes was identified in seven registered trial records (7/88, 8.0%), and 14 registered trial records (14/88, 15.9%) had the following changes: changes of study arms, sample size, blinding, controls and inclusion/exclusion criteria.

## Discussion

To assess outcome reporting bias, we identified a sample of 96 articles of acupuncture trials. Among them, 33.3% (32/96) articles showed clear discrepancies between the registered primary outcomes and the published primary outcomes. The inconsistency was addressed in 13 articles published in those journals requiring trial registration, and 5 of 13 articles were published in high-impact-factor journals. Furthermore, the other inconsistencies between the registry records and subsequent publications involved the inclusion criteria (54.7%), exclusion criteria (47.9%) and control interventions (22.9%). Selective outcome reporting has received widespread recognition. In previous studies, inconsistencies of primary outcomes between the registered records and corresponding publications in Western medicine varied from 18% and 49% [22-24].

Prospective trial registration provides a good opportunity for the editors and peer reviewers to evaluate outcome reporting bias and other deviations between the registered records and subsequent publications. However, we found some evidence of selective outcome reporting in 45.1% of the articles and other discrepancies between the published articles and the registered records in terms of inclusion/exclusion criteria, blinding, sample size, etc. These results highlight that editors and peer reviewers may not cross-check the consistency between the submitted manuscript and the registered records to identify any discrepancies, and to further limit an outcome reporting bias, even though they had a chance to access the registered records. A previous qualitative study of editors’ views on trial registration and publication bias showed that 11 of 15 journals required trial registration as a requisite for publication, but most editors seldom check whether the trial is adequately registered, and some editors did not even enforce trial registration [25].

This study also demonstrated that the trial registration number was unavailable in 36 published reports. Our finding is consistent with previous research that also indicated poor reporting of the registration number in published reports [26]. This may hamper editors and peer reviewers in accessing the registration records and to further assess the selective outcome reporting [23]. Accordingly, an accurate trial registration number should be provided in the publications. Then, the editors and peer reviewers should systematically check the consistency between the submitted manuscript and registration records, especially regarding the adequate reporting of important items such as the primary outcome.

As highlighted in our results, which confirmed the earlier findings [20,21], changes in the primary outcomes, as specified in the publications, from those specified in the registration records favored statistically significant over nonsignificant results. Such changes pose a threat to the reliability of results and further preclude clinicians and policymakers from making correct clinical decisions. Possible reasons for the discrepancies include the following: a preference for primary outcomes with statistical significance to increase the opportunity of the trial for publication and to support the use of the treatment [23,27].

The main purpose of prospective registration of clinical trials is to enhance the transparency of the clinical trial and provide access to the trial protocol [6,8]. If the trial is retrospectively registered, the meaning of the trial registration is lost; if information in registries is insufficiently detailed, with some items not registered or inadequately registered, this information will be useless for comparing the consistency between the registered records and subsequent publications. Therefore, adequate registration is of great significance to safeguard against publication bias [21]. However, our study showed that the proportion of prospective registered trials was still considerably low even with the introduction of the ICJME initiative policy implementation; registered information differed widely among trials; essential information such as randomization and blinding was poorly registered in the registries. Hence, the principal investigators and sponsors have the responsibility to adequately register a clinical trial. Furthermore, greater efforts should be made to improve the quality control processes of trial registries [21].

Although more than half of journals have required trial registration following the ICMJE guidelines, some journals use ambiguous language (i.e., encouragement of the trial registration) in their instruction to authors. We found similar results in previous studies [22,28]. A lack of submission guideline clarity might contribute to explaining inadequate trial registration and poor reporting of registration numbers.

We acknowledge that our study has several potential limitations. First, only trials indicated as having a ‘completed’ status in registries were searched for publications. A recent study indicated that many authors seemed to forget to change the status of their study to being completed, even among published studies [29]. Therefore, those trials in the registries where the status was not listed as ‘completed’ but that had been completed may not have been searched for and included in this study, which may have caused a bias.

Second, only PubMed and three Chinese databases were searched for published reports on acupuncture; therefore, some trials that have been published and included in other databases may have been missed.

## Conclusions

Although trial registration is now the rule, we find that prospective registration for RCTs on acupuncture is insufficient, selective outcome reporting is prevalent, and the change of primary outcomes is intended to favor statistical significance. These discrepancies in outcome reporting may lead to biased and misleading results of RCTs on acupuncture. To ensure publication of reliable and unbiased results, further promotion and implementation of trial registration are still needed.

## Additional files

Additional file 1: Table S1. Top ten journals publishing the included articles of acupuncture ranked by 2013 impact factor (IF) in descending order. Additional file 2: Table S2. Sample size of included articles.

## Abbreviations

ICMJE: International Committee of Medical Journal Editors
ICTRP: International Clinical Trials Registry Platform
RCTs: randomized clinical trials
TCM: traditional Chinese medicine
ANZCTR: Australian New Zealand Clinical Trials Registry
ReBec: Brazilian Clinical Trials Registry
ChiCTR: Chinese Clinical Trial Register
CRiS: Clinical Research Information Service
CTRI: Clinical Trials Registry-India
RPCEC: Cuban Public Registry of Clinical Trials
EU-CTR: EU Clinical Trials Register
DRKS: German Clinical Trials Register
ISRCTN: International Standard Randomized Controlled Trial Number Register
IRCT: Iranian Registry of Clinical Trials
JPRN: Japan Primary Registries Network
PACTR: Pan African Clinical Trial Registry
NTR: the Netherlands National Trial Register
SLCTR: Sri Lanka Clinical Trials Registry
ICD-10: International Statistical Classification of Diseases and Related Health Problems 10^th^ Revision
