# Supplementary material for: Empirical evidence for outcome reporting bias in randomized clinical trials of acupuncture: comparison of registered records and subsequent publications
Source: Trials. 2015 Jan 27;16:28. doi: 10.1186/s13063-014-0545-5 (PMC4320495; doi:10.1186/s13063-014-0545-5)
Supplement: Additional file 1: Table S1. — Top ten journals publishing the included articles of acupuncture ranked by 2013 impact factor (IF) in descending order. [file 13063_2014_545_MOESM1_ESM.docx]

**Supplement Table 1.** **Top 10 journals publishing the included articles of acupuncture ranked by 2013 impact factor (IF) in descending order**

| **Rank** | **Abbreviated Journal Title** | **Full Journal Title** | **2013-IF** | **5-year IF** | **No. of articles n (%)** |
| --- | --- | --- | --- | --- | --- |
| 1 | LANCET | LANCET | 39.207 | 39.315 | 1(1.0) |
| 2 | J CLIN ONCOL | JOURNAL OF CLINICAL ONCOLOGY | 17.879 | 17.158 | 3(3.1) |
| 3 | BMJ | BRITTISH MEDICAL JOURNAL | 16.378 | 16.122 | 5(5.2) |
| 4 | ANN INTERN MED | ANNALS OF INTERNAL MEDICINE | 16.104 | 16.482 | 3(3.1) |
| 5 | PLOS MED | PLOS MEDICINE | 14.000 | 17.945 | 1(1.0) |
| 6 | GASTROENTEROLOGY | GASTROENTEROLOGY | 13.926 | 12.951 | 3(3.1) |
| 7 | ANN ONCOL | ANNALS OF ONCOLOGY | 6.578 | 6.277 | 1(1.0) |
| 8 | ALLERGY | ALLERGY | 5.995 | 5.953 | 2(2.1) |
| 9 | Pain | Pain | 5.836 | 6.341 | 1(1.0) |
| 10 | CAN MED ASSOC J | CANADIAN MEDICAL ASSOCIATION JOURNAL | 5.808 | 7.232 | 2(2.1) |
