# Supplementary material for: Empirical evidence for outcome reporting bias in randomized clinical trials of acupuncture: comparison of registered records and subsequent publications
Source: Trials. 2015 Jan 27;16:28. doi: 10.1186/s13063-014-0545-5 (PMC4320495; doi:10.1186/s13063-014-0545-5)
Supplement: Additional file 2: Table S2. — Sample size of included articles. [file 13063_2014_545_MOESM2_ESM.docx]

**Supplement 2. Sample size of included articles**

| **Sample** | **No. of articles** | **Proportion (%)** |
| --- | --- | --- |
| 1-10 | 1 | 1.0 |
| 11-20 | 4 | 4.2 |
| 21-50 | 20 | 20.8 |
| 51-100 | 30 | 31.3 |
| 101-200 | 17 | 17.7 |
| 201-500 | 18 | 18.8 |
| 501-1000 | 6 | 6.2 |
| Total | 96 | 100 |
